# First identification and genotyping of *Enterocytozoon bieneusi* and *Encephalitozoon* spp. in pet rabbits in China

**DOI:** 10.1186/s12917-020-02434-z

**Published:** 2020-06-22

**Authors:** Lei Deng, Yijun Chai, Leiqiong Xiang, Wuyou Wang, Ziyao Zhou, Haifeng Liu, Zhijun Zhong, Hualin Fu, Guangneng Peng

**Affiliations:** The Key Laboratory of Animal Disease and Human Health of Sichuan Province, College of Veterinary Medicine, Sichuan Agricultural University, Chengdu, 611130 Sichuan China

**Keywords:** Microsporidia, Rabbits, ITS, Microsporidiosis, China

## Abstract

**Background:**

Microsporidia are common opportunistic parasites in humans and animals, including rabbits. However, only limited epidemiology data concern about the prevalence and molecular characterization of *Enterocytozoon bieneusi* and *Encephalitozoon* spp. in rabbits. This study is the first detection and genotyping of Microsporidia in pet rabbits in China.

**Results:**

A total of 584 faecal specimens were collected from rabbits in pet shops from four cities in Sichuan province, China. The overall prevalence of microsporidia infection was 24.8% by nested PCR targeting the internal transcribed spacer (ITS) region of *E. bieneusi* and *Encephalitozoon* spp. respectively. *E. bieneusi* was the most common species (*n* = 90, 15.4%), followed by *Encephalitozoon cuniculi* (*n* = 34, 5.8%) and *Encephalitozoon intestinalis* (*n* = 16, 2.7%). Mixed infections (*E. bieneusi* and *E. cuniculi*) were detected in five another rabbits (0.9%). Statistically significant differences in the prevalence of microsporidia were observed among different cities (χ^2^ = 38.376, df = 3, *P* < 0.01) and the rabbits older than 1 year were more likely to harbour microsporidia infections (χ^2^ = 9.018, df = 2, *P* < 0.05). Eleven distinct genotypes of *E. bieneusi* were obtained, including five known (SC02, I, N, J, CHY1) and six novel genotypes (SCR01, SCR02, SCR04 to SCR07). SC02 was the most prevalent genotype in all tested cities (43.3%, 39/90). Phylogenetic analysis showed that these genotypes were clustered into group 1–3 and group 10. Meanwhile, two genotypes (I and II) were identified by sequence analysis of the ITS region of *E. cuniculi*.

**Conclusion:**

To the best of our knowledge, this is the first report of microsporidia infection in pet rabbits in China. Genotype SC02 and four novel genotypes were classified into potential zoonotic group 1, suggesting that pet rabbits may cause microsporidiosis in humans through zoonotic transmissions. These findings provide preliminary reference data for monitoring microsporidia infections in pet rabbits and humans.

## Background

Microsporidia, as obligate intracellular parasites and classified as fungi, are emerging opportunistic pathogens that can infect many invertebrates and vertebrates, including humans and rabbits [1]. To date, the phylum Microsporidia consists of at least 200 genera and 1500 species, of which 17 microsporidia species have been detected in humans [2, 3]. Among them, *Enterocytozoon bieneusi* and *Encephalitozoon* spp. (including *E. cuniculi*, *E. hellem*, and *E. intestinalis*) are the four most common microsporidia species that infect humans, domestic animals, and wildlife [4, 5]. Microsporidia are often considered as a major pathogen of chronic diarrhea in severely immune-compromised patients, such as AIDS patients and solid organ transplant recipients [6]. Besides, the discovery of microsporidia in water sources intended for human consumption has made it a Category B Priority Pathogen listed by the National Institutes of Health (NIH), and it has also been listed by the United States Environmental Protection Agency (EPA) as a microbial pollutant potentially causing waterborne outbreaks [1, 7].

More than 470 *E. bieneusi* genotypes have been identified in humans and animals based on sequence analysis of the internal transcribed spacer (ITS) region of ribosomal RNA (rRNA), with more new genotypes continually being found [8]. Some of these genotypes are considered to be host-specific, while others have zoonotic potential (e.g., SC02, D, EbpC, J, I and Type IV) [9]. Based on the number of 5′-GTTT-3′ repeats in the ITS sequence, four distinct genotypes (genotype I -IV) of *E. cuniculi* have been identified [10, 11]. *E. hellem* also has four genotypes (1 to 4) by analysis of the ITS sequence [12]. However, no intraspecific variation in the ITS sequence of *E. intestinalis* has been detected thus far.

In China, *E. bieneusi* and *Encephalitozoon* spp. have been identified in a broad range of wild and domestic animal hosts, including mammals, reptiles, and birds [13]. Rabbits have been reported to harbor various zoonotic species (e.g., *Cryptosporidium*, *Giardia*, Microsporidia, and *Toxoplasma gondii*) and are considered to be a potential source of human infections [14, 15]. However, only limited information is available on *E. bieneusi* and *Encephalitozoon* spp. infection in pet rabbits in China. Moreover, pet rabbits are popular companions and their close relationship with humans may represent a still not completely understood health threat. Therefore, the purpose of the present study was to determine the prevalence and molecular characteristics of microsporidia in faecal samples of pet rabbits, as well as to assess the zoonotic potential of these pathogens.

## Results

### Prevalence of E. bieneusi and Encephalitozoon spp.

A total of 584 faecal samples of pet rabbits from 12 pet shops in four cities in Sichuan province of southwestern of China were examined using molecular methods. The specific primers for *E. bieneusi* and *Encephalitozoon* spp. were used to determine the presence of microsporidia. In total, 24.8% (145/584) of the rabbits were found infected with microsporidians. Single-species infection was detected in 90 rabbits (15.4%) for *E. bieneusi*. 34 (5.8%) and 16 (2.7%) *E. cuniculi* and *E. intestinalis* mono-infections were identified respectively (Table 1). *Encephalitozoon hellem* was not identified in the surveyed population. In addition, 5 samples were identified as coinfection (0.9%) (Table 1).

**Table 1:** The prevalence and genotype distribution of microsporidia in pet rabbits in southwestern China

Location	No. positive/No. tested (%)	PCR positive						
		*E. bieneusi*		*E. cuniculi*		*E. intestinalis*	Mixed infection	
		No. (%)	Genotype (n)	No. (%)	Genotype (n)	No. (%)	No. (%)	Genotype (n)
Chengdu	70/313 (22.4)	44 (14.1)	SC02 (21), I (6), J (5), N, (8), SCR01 (1), SCR02 (1), SCR06 (2)	17 (5.4)	I (11), II (6)	7 (2.2)	2 (0.6)	SC02 + I (1), J + I (1)
Luzhou	9/75 (12.0)	7 (9.3)	SC02 (3), I (3), J (1)	0	0	2 (2.7)	0	0
Yaan	50/106 (47.2)	25 (23.6)	SC02 (8), I (7), I (5), N (3), SCR04 (1), CHY1 (1)	15 (14.2)	I (8), II (7)	7 (6.6)	3 (2.8)	SC02 + I (2), J + II (1)
Ziyang	16/90 (17.8)	14 (15.6)	SC02 (7), I (2), SCR05 (2), SCR07 (3)	2 (2.2)	II (2)	0	0	0
Total	145/584 (24.8)	90 (15.4)	SC02 (39), I (21), N (13), J (6), CHY1 (1), SCR01 (1), SCR02 (1), SCR04 (1), SCR05 (2), SCR06 (2), SCR07 (3)	34 (5.8)	I (19), II (15)	16 (2.7)	5 (0.9)	SC02 + I (3), J + II (1), J + I (1)

Note: genotype CHY1 is a synonym of genotype S7

Microsporidia-positive samples were detected in all tested cities, and the prevalence of microsporidia ranged from 12.0 to 47.2% (χ^2^ = 38.376, df = 3, *P* < 0.01) (Table 1). The Dutch breed (28.3%) had a higher susceptibility to microsporidia infection than other breeds; however, the differences among pet rabbit breeds were not significant (χ^2^ = 3.140, df = 5, *P* > 0.05) **(**Table 2**)**. The prevalence in rabbits ≥12 months of age was significantly higher (35.9%) than those at 6–12 months of age (21.2%) (χ^2^ = 9.018, df = 2, *P* < 0.05). However, there were no significant differences in the prevalence between males (25.7%) and females (23.8%) (χ^2^ = 0.289, df = 1, *P* > 0.05).

**Table 2:** The prevalence of microsporidia in pet rabbits by breed, age, and sex in southwestern China

Group	No. of tested	No. of positive	Prevalence (%)	95% CI	*P*-value
Breed					
Dutch rabbit	205	58	28.3	22.1–34.5	0.68
New Zealand Rabbit	97	24	24.7	16.2–33.3	
Lop ear rabbit	94	20	21.3	13.0–29.6	
Pygmy rabbit	80	18	22.5	13.3–31.7	
Pearl rabbit	65	17	26.2	15.5–36.8	
Lion head rabbit	43	8	18.6	7.0–30.2	
Age (months)					
≤ 6	165	41	24.8	18.3–31.4	0.01
6–12	316	67	21.2	16.7–25.7	
≥ 12	103	37	35.9	26.7–45.2	
Sex					
Male	319	82	25.7	20.9–30.5	0.59
Female	265	63	23.8	18.6–28.9	

### Molecular characterization of *E. bieneusi* and *Encephalitozoon* spp.

Analysis of the ITS sequences of the *E. bieneusi*-positive samples revealed the presence of 11 distinct genotypes, including five known (SC02, I, N, J, CHY1) and six novel genotypes (SCR01, SCR02, SCR04 to SCR07). Genotype SC02 was the most prevalent (43.3%, 39/90) and displayed 100% homology with the previously published GenBank Accession No. KY950533 (from a giant panda in China). Followed by genotype I (23.3%, 21/90), which was identical to the sequence AF135836 (from cattle in Germany). Thirteen samples were characterized as genotype N and displayed 100% homology with the GenBank Accession No. AF267144, and six samples as genotype J identical to GenBank Accession No. AF135837. Genotype CHY1 showed 100% homology with the GenBank Accession No. KT267289 (from a yak in China).

For the novel genotypes of *E. bieneusi*, genotypes SCR01, SCR02 and SCR04 were found to have two, four, and five single nucleotides polymorphisms (SNPs), respectively, when compared to genotype SC02 (accession No. KY950533). Genotype SCR05 had six SNPs when compared to genotype XJH2 (accession No. KU194604; from a horse in China). Further, genotype SCR06 had seven SNPs in comparison to genotype J (GenBank Accession No. AF135837) and genotype SCR07 had eight SNPs when compared to genotype N (GenBank Accession No. AF267144). The genetic polymorphism of the novel genotypes within the 243 bp of the ITS sequence are presented in Additional file 2.

Sequence analysis of 34 isolates of *E. cuniculi* revealed that 19 isolates were identical with genotype I (GenBank Accession No. KJ469979 from *Gorilla beringei beringei* in Rwanda) and 14 isolates were identical to genotype II (GenBank Accession No. GU213880 from a cat in Austria). The sequence of *E. intestinalis* showed 100% identity with the deposited sequences in the GenBank (Accession No. GQ408912 from a patient with HIV in Russia).

Regarding to co-infections, three rabbits showed mixed infections of genotype SC02 of *E. bieneusi* and genotype I of *E. cuniculi*. One animal showed co-infections of genotype J of *E. bieneusi* and genotype II of *E. cuniculi*. Further, a co-infection of genotype J of *E. bieneusi* and genotype I of *E. cuniculi* was observed in one rabbit.

### Phylogenetic relationship of *E. bieneusi*

**Fig. 1:**
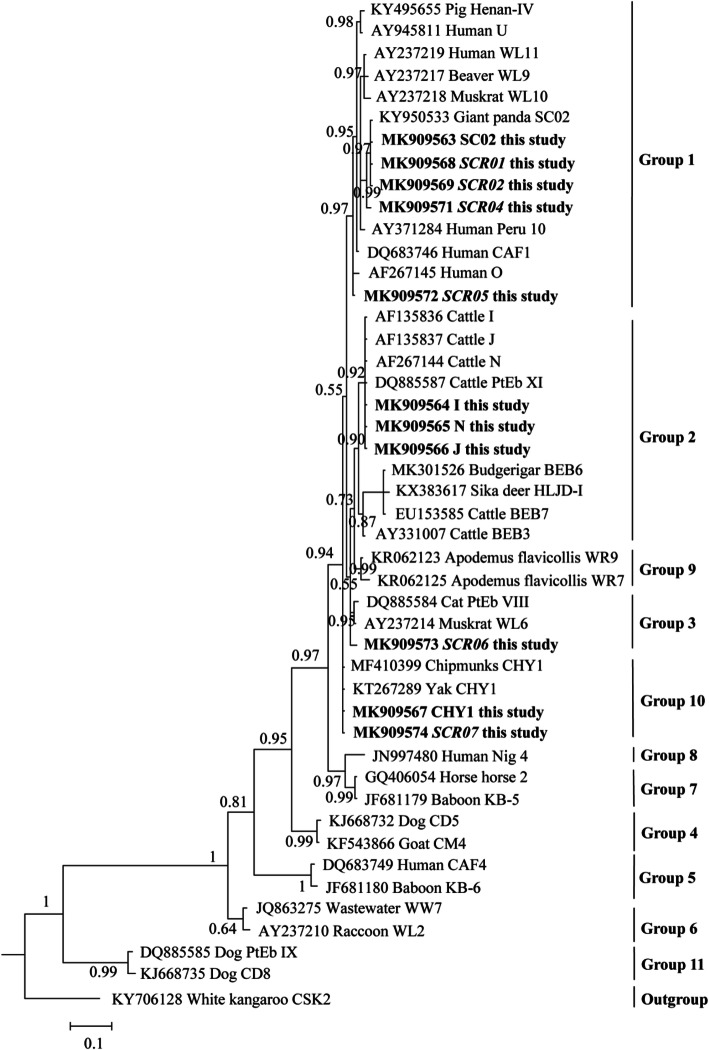
Phylogenetic analysis of internal transcribed spacer of ribosomal RNA of *E. bieneusi* by Bayesian inference. Statistically significant posterior probabilities are indicated on the branches. Each sequence is identified by its accession number, host and genotype designation. Known and novel *E. bieneusi* genotypes identified in the present study are indicated by bold, and the novel genotypes are shown by italic. The *E. bieneusi* genotype CSK2 (KY706128) from white kangaroo was used as the outgroup

The ITS sequence data for the 11 distinct genotypes identified in this study were included in a phylogenetic analysis, together with sequences representing 11 established groups of *E. bieneusi*. Five genotypes (SC02, SCR01, SCR02, SCR04, and SCR05) were classified into zoonotic potential group 1. Three known genotypes (I, N, and J) were clustered into group2. Genotype SCR06 belonged to group 3. Genotypes CHY1 and SCR07 were classified into group 10 (Fig. 1).

## Discussion

Although *E. bieneusi* and *Encephalitozoon* spp. mainly cause infections and potentially life-threatening diseases in individuals with immune deficiency, the routes of transmission as well as source of infection are not fully understood. Environmentally-resistant microsporidial spores of human and animal origin have been consistently found in surface waters, raising concerns about waterborne outbreaks [16]. So far, the increasing number of researchers have focused on animal microsporidiosis to explain possible sources of human microsporidiosis in the past few years, but few studies have been conducted on pet rabbits in close contact with humans. To the best of our knowledge, this is the first molecular identification of *E. bieneusi* and *Encephalitozoon* spp. in pet rabbits in China.

In the present study, we found an age-related infection pattern, with animals > 1 year being significantly more infected by microsporidia than younger ones (χ2 = 9.018, df = 2, *p* < 0.05), which is consistent with previous findings in domestic rabbits in northeastern China [17]. In addition, similar findings were also demonstrated in other animals, such as dogs, donkeys, and foxes [18–20]. These findings might suggest that the parasite tends to accumulate with age and, therefore, could behave as a commensal rather than a pathogen.

*E. bieneusi* has been detected in a wide range of animals, such as macaques, pigs, cattle, horses, dogs, cats, raccoons, foxes, llamas, pigeons, and farmed rabbits [18, 21, 22]. The prevalence of *E. bieneusi* was 15.4% (90/584), which is similar with the previously reported in Rex rabbits (14.7%, 22/150) and other pets in China [23–25], but higher than that most previously reported rates from Chinese provinces [26, 27] (Table 3). The differences in *E. bieneusi* infection rates may be due to the fact that rabbits are more susceptible to infection than dogs and cats. Genotype SC02 has been identified in a wide range of animals in China, including nonhuman primates, wild boars, horses, giant pandas, and squirrels [8]. Genotype SC02 was predominant in the present study, which differs from the findings of previous studies in Rex rabbits in Heilongjiang province [23], and in domestic rabbits in Xinjiang [26]. Genotypes N, I, and J were originally detected in cattle, but recently these genotypes have also been detected in non-human primates, donkey, cats, sika deer, birds, and humans [21]. Genotype CHY1 is a synonym of genotype S7, which was identified in a patient in the Netherlands [33], as well as in yaks [34], in pet chipmunks [25], and more recently, in pet rats [35]. Notably, Cama et al. reported a possible transmission of *E. bieneusi* between children and guinea pigs in the same household, suggesting the possibility of zoonotic transmission between human and pet animal [36]. These data suggest that these genotypes have a broad host range and zoonotic potential.

**Table 3:** The prevalence and genotypes of *Enterocytozoon bieneusi* in rabbits and various pets in China

Province	Host	No. positive/No. examined (%)	Genotype (n)	References
Jilin	Rabbits	3/174 (1.7)	D (3)	[17]
Liaoning	Rabbits	1/136 (0.7)	D (1)	[17]
Heilongjiang	Rex rabbits	22/150 (14.7)	CHN-RD1 (12), D (3), Type IV (2), I (1), Peru6 (1), CHN-RR1 (1), CHN-RR2 (1), CHN-RR3 (1)	[23]
Xinjiang	Rabbits	9/321 (2.8)	J (5), BEB8 (3), Type IV (1)	[26]
Jilin	Dog	2/26 (7.8)	CHN5 (1), CHN6 (1)	[28]
Henan	Dog	13/133 (9.8)	PtEbIX (3), CM1 (2), D (2), Peru8 (1), type IV (1), CD2 (1), CD6 (1), CD7 (2)	[20]
Shanxi	Dog	6/30 (20.0)	PtEbIX (2), EbpC (1), CD8 (2), CD9 (1)	[20]
Chongqing	Dog	4/34 (11.8)	PtEbIX (2), CD8 (2)	[20]
Heilongjiang	Dog	18/267 (6.7)	D (1), EbpC (2), NED1 (1), NED2 (1); PtEb IX (14), NED3 (1), NED4 (1) (mix infection)	[29]
Shanghai	Dog	29/485 (6.0)	PtEb IX (28), D (1)	[30]
Henan	Cat	11/96 (11.5)	D (3), BEB6 (2), I (1), PtEbIX (1), CC1 (1), CC2 (1), CC3 (1), CC4 (1)	[20]
Heilongjiang	Cat	3/52 (5.8)	D (2), type IV (1)	[29]
Shanghai	Cat	9/160 (5.6)	Type IV (5), D (4)	[30]
Henan	Chinchilla	4/102 (3.9)	D (2), BEB6 (2)	[31]
Beijing	Chinchilla	1/26 (3.8)	BEB6 (1)	[31]
Sichuan	Pet birds	97/387 (25.1)	D (41), SC02 (29), BEB6 (14), CHB1 (4), MJ5 (3), SCB-I (3), SCB-II (1), SCB-III (2)	[32]
Sichuan	Various pet rabbits	90/584 (15.4)	SC02 (39), I (21), N (13), J (6), CHY1 (1), SCR01 (1), SCR02 (1), SCR04 (1), SCR05 (2), SCR06 (2), SCR07 (3)	This study

Phylogenetic analysis was conducted to reveal the relationship and genetic diversity between the 11 genotypes identified in the present study and other representative known genotypes. The known genotype SC02 and four novel genotypes (SCR01, SCR02, SCR04, SCR05) belonged to group 1, which has been considered has significant zoonotic importance [3]. Group 1 consists of mostly genotypes from humans and genotypes from a wide range of animals, including nonhuman primates, porcines, bovines, cats, dogs, equines, carnivores, rodents, lagomorphs, marsupials, birds, and some rare hosts (e.g., bat, hippo, snake, muskrat, vole, beaver) [8, 37]. Although there is no clear evidence that human infection with *E. bieneusi* is related to pet rabbits, direct contact with infected rabbits or drinking contaminated water by spore of microsporidia is considered to be an important risk factor for the spread of microsporidiosis.

The prevalence of *E. cuniculi* in the present study was 5.8%, which is lower than that in rabbits in Taiwan (67.8%) [38], Italy (67.2%) [39], and Austria (58.5%) [40]. Based on the ITS region of rRNA gene, we identified genotypes I and II of *E. cuniculi* in pet rabbits. Type I primarily infects rabbits, and infections have been reported in the USA, Australia, and Europe [41]. Type II has only been confirmed in pigeons in Iran, in waters in Switzerland, and in pigs in Europe [42]. This is the first study of *E. intestinalis* infection in rabbits, reporting a prevalence of 2.7%. Due to the lack of data regarding the prevalence of *E. cuniculi* and *E. intestinalis* in humans and other hosts in the investigated areas, the actual infection sources and transmission routes were not elucidated in the present study.

## Conclusions

This is the first report of the *E. bieneusi* and *Encephalitozoon* spp. in pet rabbits in China. The overall prevalence of microsporidia in pet rabbits was 24.8% and some known zoonotic genotypes were identified, suggesting pet rabbits may play a role in the transmission of these pathogens to humans and other animals. These findings extend.

the knowledge of the microsporidia distribution among pet rabbits and provide fundamental data for controlling microsporidiosis in pet rabbits and humans.

## Methods

### Collection of faecal samples

Between July 2017 and January 2019, a total of 584 faecal samples were collected from rabbits in 12 pet shops located in four cities of Sichuan province, southwestern China. The pet shops were randomly selected according to the estimated number of pet shops per area. All tested pet shops only raised rabbits and served as suppliers of rabbits to other pet shops. Before signing a formal consent, the manager of each pet shop was informed about the study purpose and procedures. Only one sample was collected from each animal. Faecal samples were collected from the bottom of each cage and then individually placed into 30 mL sterile plastic containers with ice packs. All samples were transported to the laboratory within 24 h. The age, sex, source, and health condition of each rabbits were recorded at the sampling site. All animals were healthy and none showed any clinical signs of gastrointestinal disease at the time of sampling.

### DNA extraction

All faecal samples were suspended in distilled water, and the suspension was then passed through a 250 μm pore size wire mesh sieve. The filtrate was centrifuged at 1500×g for 10 min. Genomic DNA was extracted from ~ 200 mg of each processed faecal sample using QIAamp® Stool Mini Kit (Qiagen, Hilden, Germany) according to the manufacturer’s instructions. DNA was eluted in 50 μl of nuclease-free water (Promega, Madison, USA), and acquired DNA was stored at − 20 °C until use.

### PCR amplification and sequence analysis

*E. bieneusi* genotypes were determined by nested PCR of the ITS region of rRNA as described by Sulaiman et al. [43]. *Encephalitozoon* spp. ITS was amplified using MSP-1 and MSP-2A as the outer primer pair, and MSP-3 and MSP-4A as the inner primer pair [44, 45]. PCR amplification primers and cycling conditions in this study are presented in Additional file **1****.** PCR was performed with a 50 μl volume containing 25 μl *Taq* PCR Master Mix (Sangon Biotech Co., Ltd., Shanghai, China), 2 μl each primer (0.4 μM), 1 μl of each DNA sample, 1.5 μl MgCl_2_ (25 mM) and nuclease-free water up to desired volume. Positive and negative controls were included in all the PCR tests. The secondary PCR products were examined by 2% agarose gel electrophoresis and visualized after ethidium bromide staining.

Positive secondary PCR products were sent to Life Technologies for sequencing with an ABI 3730 DNA Analyzer using the BigDye® Terminator v3.1 cycle sequencing kit (Applied Biosystems, Foster City, CA, USA) at the Sangon Biotech Company (Chengdu, China). The sequences were edited and aligned using ClustalW (http://www.ebi.ac.uk/Tools/msa/clustalw2/) and compared with reference sequences from GenBank using BLAST tool (http://blast.ncbi.nlm.nih.gov/Blast.cgi). The accuracy of the sequences of the novel genotypes was confirmed by resequencing the obtained amplicons.

### Phylogenetic and statistical analyses

Bayesian inference (BI) and Monte Carlo Markov Chain (MCMC) methods were used to construct the phylogenetic tree in MrBayes version 3.2.5 [46]. Akaike Information Criteria (AIC) test in ModelFinder was used for evaluating the substitution model that best fit the dataset [47]. The posterior probability (pp) values were calculated by running 2,000,000 generations. A 50% majority-rule consensus tree was constructed from the final 75% of the trees generated by BI. Analyses were run three times to ensure convergence and insensitivity to priors.

Data were analyzed using SPSS statistical software (version 22) and the Chi-square test was used to detect significant differences. *P*-values < 0.05 were considered statistically significant.

## Supplementary information

**Additional file 1: Table S1.** Primer sequences, fragment lengths and annealing temperatures used in this study.

**Additional file 2: Figure S1.** Sequence variation in the ITS region of the rRNA gene of *Enterocytozoon bieneusi* isolates from pet rabbits. The ITS sequences of five known genotypes (SC02, I, N, J, and CHY1) and the six novel genotypes (SCR01, SCR02, SCR04 to SCR07), identified in this study, were aligned with each other.

## Data Availability

Representative nucleotide sequences were deposited in GenBank with the following accession numbers: MK909563- MK909569 and MK909571- MK909574 (*E. bieneusi*), MK909577 (*E. intestinalis*), MK909562 and MN749308 (*E. cuniculi*).

## Abbreviation

ITS: Internal transcribed spacer
*E. bieneusi*: *Enterocytozoon bieneusi*
*E. cuniculi*: *Encephalitozoon cuniculi*
*E. intestinalis*: *Encephalitozoon intestinalis*
*E. hellem*: *Encephalitozoon hellem*
NIH: National Institutes of Health
EPA: Environmental Protection Agency
rRNA: Ribosomal RNA
BI: Bayesian inference
MCMC: Monte Carlo Markov Chain
AIC: Akaike Information Criteria
pp.: Posterior probability
