# Supplementary material for: First identification and genotyping of Enterocytozoon bieneusi and Encephalitozoon spp. in pet rabbits in China
Source: BMC Vet Res. 2020 Jun 22;16:212. doi: 10.1186/s12917-020-02434-z (PMC7310219; doi:10.1186/s12917-020-02434-z)
Supplement: Supplementary file 1 — Additional file 1: Table S1. Primer sequences, fragment lengths and annealing temperatures used in this study. [file 12917_2020_2434_MOESM1_ESM.docx]

**Table S1** Primer sequences, fragment lengths and annealing temperatures used in this study.

| Gene | Primer | Sequence (5'-3') | Annealing temperature (˚C) | Fragment length (bp) | Reference |
| --- | --- | --- | --- | --- | --- |
| *E. bieneusi* ITS | AL4037 | GATGGTCATAGGGATGAAGAGCTT | 55 | ~392 | Sulaiman et al., 2003 |
|  | AL4039 | AATACAGGATCACTTGGATCCGT |  |  |  |
|  | AL4038 | AGGGATGAAGAGCTTCGGCTCTG |  |  |  |
|  | AL4040 | AATATCCCTAATACAGGATCACT |  |  |  |
| *Encephalitozoon* spp. ITS | MSP-1 | TGAATG(G/T)GTCCCTGT | 55 | ~300 | Franzen and Müller, 1999; Katzwinkel-Wladarsch et al., 1996 |
|  | MSP-2A | TCACTCGCCGCTACT |  |  |  |
|  | MSP-3 | GGAATTCACACCGCCCGTC(A/G)(C/T)TAT |  |  |  |
|  | MSP-4A | CCAAGCTTATGCTTAAGT(C/T)(A/C)AA(A/G)G GGT |  |  |  |

Sulaiman, I.M., Fayer, R., Lal, A.A., Trout, J.M., Rd, S.F., and Xiao, L. (2003). Molecular Characterization of Microsporidia Indicates that Wild Mammals Harbor Host-Adapted *Enterocytozoon* spp. as well as Human-Pathogenic *Enterocytozoon bieneusi*. Appl Environ Microbiol. 69, 4495. doi: 10.1128/aem.69.8.4495-4501.2003

Franzen, C., and Müller, A. (1999). Molecular techniques for detection, species differentiation, and phylogenetic analysis of microsporidia. Clin Microbiol Rev. 12, 243-285. doi: 10.1128/CMR.12.2.243

Katzwinkel-Wladarsch, S., Lieb, M., Helse, W., Löscher, T., and Rinder, H. (1996). Direct amplification and species determination of microsporidian DNA from stool specimens. Trop Med Int Heal. 1, 373-378. doi: 10.1046/j.1365-3156.1996.d01-51.x
