# Supplementary material for: First identification and genotyping of Enterocytozoon bieneusi and Encephalitozoon spp. in pet rabbits in China
Source: BMC Vet Res. 2020 Jun 22;16:212. doi: 10.1186/s12917-020-02434-z (PMC7310219; doi:10.1186/s12917-020-02434-z)

**Figure S1.** Sequence variation in the ITS region of the rRNA gene of *Enterocytozoon bieneusi* isolates from pet rabbits. The ITS sequences of five known genotypes (SC02, I, N, J, and CHY1) and the six novel genotypes (SCR01, SCR02, SCR04 to SCR07), identified in this study, were aligned with each other.


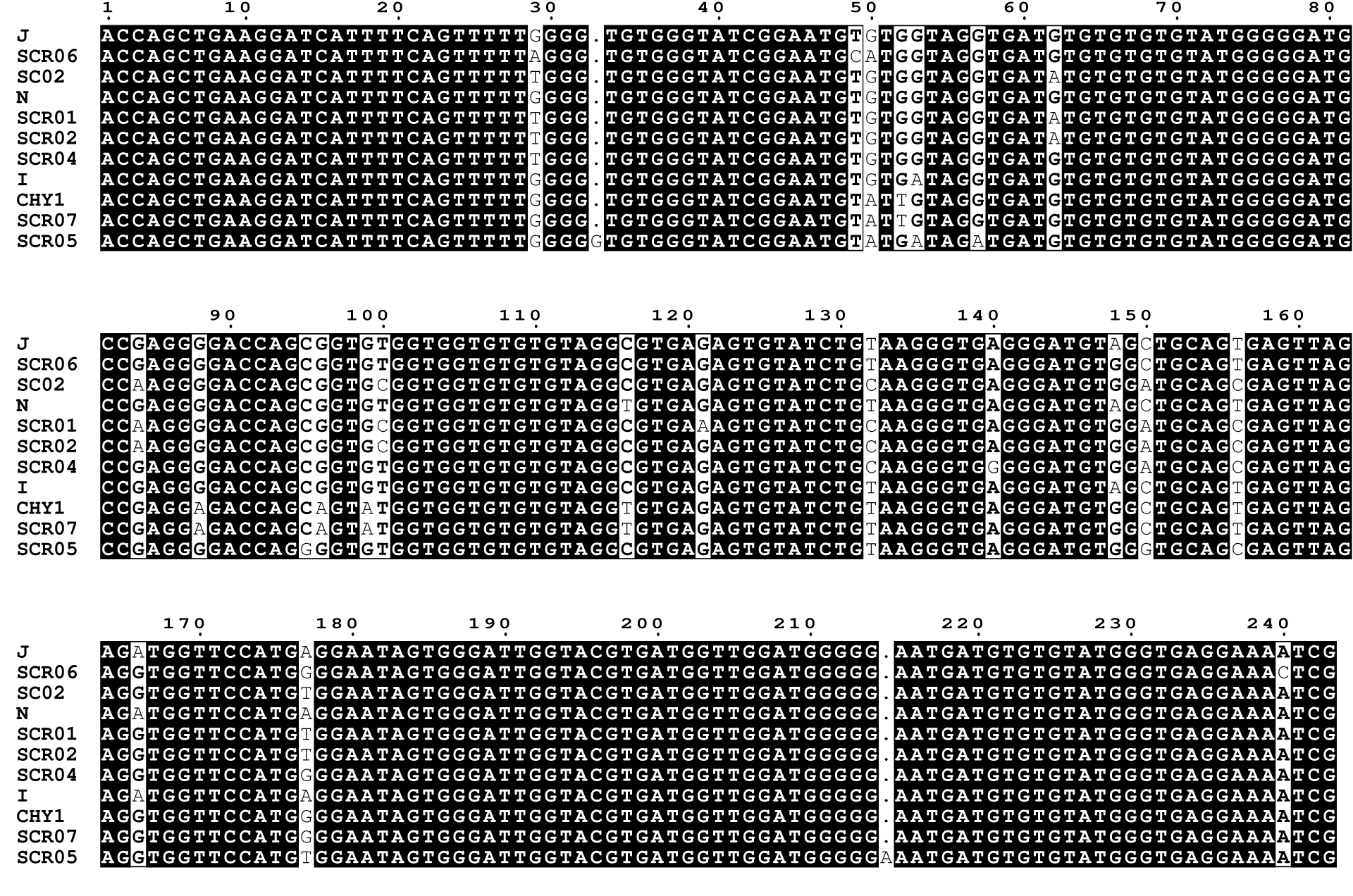

Supplement: Supplementary file 2 — Additional file 2: Figure S1. Sequence variation in the ITS region of the rRNA gene of Enterocytozoon bieneusi isolates from pet rabbits. The ITS sequences of five known genotypes (SC02, I, N, J, and CHY1) and the six novel genotypes (SCR01, SCR02, SCR04 to SCR07), identified in this study, were aligned with each other. [file 12917_2020_2434_MOESM2_ESM.docx]
